# Congenital nystagmus and central hypothyroidism

**DOI:** 10.1186/s13633-015-0003-5

**Published:** 2015-03-16

**Authors:** Nele Reynaert, Elke Braat, Francis de Zegher

**Affiliations:** Department of Pediatric Endocrinology, University Hospitals Leuven, Herestraat 49, 3000 Leuven, Belgium; Department of Pediatrics, University Hospitals Leuven, Leuven, Belgium

**Keywords:** Congenital hypothyroidism, Nystagmus, *FRMD7*, *IGFS1*

## Abstract

We observed a male newborn with bilateral nystagmus and central hypothyroidism without hypoprolactinemia due to a deletion of chromosome band Xq26.1q26.2, containing *FRMD7* and *IGSF1*. These two loss-of function mutations are known to cause, respectively, congenital nystagmus and the ensemble of central hypothyroidism, hypoprolactinemia and testicular enlargement. These latter two features may not yet be present in early life.

## Letter to the editor

In 1969, Schulman and Crawford reported a boy with congenital nystagmus and central hypothyroidism (“congenital, isolated TSH deficiency”) – an apparently rare and still unexplained association [1].

Recently, we observed a male newborn with bilateral nystagmus and central hypothyroidism. At term birth, he presented with an umbilical hernia, enlarged tongue and need for additional oxygen. On day 3, serum free T4 was only 0.52 ng/dL (normally ≥2.0 ng/dL), TSH elevation was limited (10.6 mU/L); L-thyroxine treatment was initiated.

Early gestation had been complicated by nuchal enlargement, which prompted a chorion biopsy that led to the identification of a 1.29 Mb deletion of chromosome band Xq26.1q26.2 [arr Xq26.1q26.2(129928356–131292675)x0]. The deleted region contains – besides five genes so far unassociated with disease (*ENOX2, ARHGA36, OR13H1, FIRRE, MST4)* – *FRMD7* and *IGSF1*, loss-of-function mutations in which are known to cause, respectively, congenital nystagmus [2] and the ensemble of central hypothyroidism, hypoprolactinemia and testicular enlargement [3]. The latter features may not yet be present in early life since the hypothyroid newborn had elevated concentrations of circulating prolactin (266 μg/L on day 3) and normal testicular volumes (2 mL by orchidometer).

In conclusion, nearly half a century after the first report on an enigmatic association of congenital nystagmus and central hypothyroidism, we identified a male newborn with the same association and a Xq26 deletion encompassing *FRMD7* and *IGSF1*.
